# Understanding the use of over-the-counter pain treatments in adolescents with chronic pain

**DOI:** 10.1080/24740527.2017.1337468

**Published:** 2017-08-04

**Authors:** Jennifer Stinson, Lauren Harris, Elizabeth Garofalo, Chitra Lalloo, Lisa Isaac, Stephen Brown, Jennifer Tyrrell, Danielle Ruskin, Fiona Campbell

**Affiliations:** a Department of Anesthesia and Pain Medicine, The Hospital for Sick Children, Toronto, Ontario, Canada; b Department of Child Health Evaluative Sciences, The Hospital for Sick Children, Toronto, Ontario, Canada; c Lawrence S. Bloomberg Faculty of Nursing, University of Toronto, Toronto, Ontario, Canada; d Department of Anaesthesia, University of Toronto, Toronto, Ontario, Canada

**Keywords:** Chronic pain, adolescent, medication, nonprescription, qualitative

## Abstract

**Background**: The prevalence of chronic pain in children and adolescents is well established. What is not well understood is how over-the-counter (OTC) oral and topical pain treatments are being used by adolescents with chronic pain, their decision making around use of these products, and how they communicate with their health care providers about their use.

**Aims**: The aim of this study was to explore the use, decision-making process, and communication about the use of OTC pain medications with health care professionals among adolescents living with chronic pain and their primary caregiver.

**Methods**: A qualitative descriptive design with semistructured, audiotaped individual interviews was undertaken with adolescents with chronic pain (*n* = 15, aged 12–18 years, mean age = 16, *SD* = 1.79) and their caregivers (*n* = 16). A convenience sample of patient–caregiver dyads was recruited from a tertiary care pediatric chronic pain clinic in Ontario.

**Results**: Interview questions focused on four topics: (1) experience with chronic pain and medication; (2) perceptions of medications and concerns with long-term consumption; (3) decision making for use of OTC medications guided mainly by a trusted source or health care professional; and (4) topical OTC medications perceived as harmless. Content analysis within these four topics uncovered two to four subthemes, which are described in detail.

**Conclusions**: An improved understanding of the prevalence of use, decision-making process around use, and how patients and their families communicate about the use of OTC pain medications with health care providers can help clinicians better personalize treatments and help adolescents with chronic pain to make sound self-care decisions.

## RÉSUMÉ

**Contexte**: La prévalence de la douleur chronique chez les enfants et les adolescents est bien établie. Toutefois, on ne comprend pas bien comment les traitements antidouleur en vente libre administrés par voie topique ou orale sont utilisés par les adolescents souffrant de douleur chronique, leur prise de décision concernant ces produits et la façon dont ils communiquent avec les prestataires de soins de santé au sujet de leur utilisation.

**But**: Étudier l’utilisation des médicaments antidouleur en vente libre, ainsi que la prise de décision et la communication avec les professionnels de la santé au sujet de l’utilisation de ces médicaments chez les adolescents souffrant de douleur chronique et leur principal prestataire de soins.

**Méthodes**: Un devis de recherche axé sur la description qualitative à l’aide d’entrevues semi-structurées individuelles a été utilisé auprès d’adolescents souffrant de douleur chronique (n = 15, âgés de 12 à 18 ans, âge moyen de 16 ans, ÉT = 1,79) et leurs prestataires de soins (n = 16). Un échantillon de commodité de dyades patient-prestataire de soins a été recruté dans une clinique de soins tertiaires en douleur chronique pédiatrique de l’Ontario.

**Résultats**: Les questions d’entrevue portaient sur quatre sujets: (1) l’expérience de la douleur chronique et la médication; (2) la perception des médicaments et les préoccupations concernant leur consommation à long terme; (3) la prise de décision concernant l’utilisation de médication en vente libre, principalement guidée par une personne de confiance ou un professionnel de la santé et (4) les médicaments en vente libre appliqués par voie topique qui sont perçus comme étant sans danger. L’analyse de contenu pour ces quatre sujets a révélé de 2 à 4 sous-thèmes, qui sont décrits en détail.

**Conclusions**: Une meilleure compréhension de la prévalence de l’utilisation des médicaments antidouleur en vente libre, ainsi que du processus de décision entourant leur utilisation et de la façon dont les patients et leur famille communiquent avec les professionnels de la santé peut aider les cliniciens à mieux personnaliser les traitements et aider les adolescents qui souffrent de douleur chronique à prendre des décisions judicieuses en matière de soins auto-administrés.

## Introduction

Chronic pain that is unrelieved or undertreated in children and adolescents can negatively impact all aspects of health-related quality of life.^1^ A recent systematic review of population-based studies found a high prevalence of chronic pain in adolescents, with rates ranging from 8% to 40%.^2^ A subset of these adolescents face moderate to severe functional limitations or disability due to their chronic pain.^3,4^ A multimodal approach to pediatric chronic pain treatment using pharmacological, physical, and psychological strategies results in the best outcomes.^5–7^ In addition to pharmacological treatments prescribed by their health care providers (HCPs), many adolescents with chronic pain often use over-the-counter (OTC) oral and topical medications (e.g., anti-inflammatories, acetaminophen, muscle relaxants). Better understanding the use of OTC medications at home could enable clinicians to better personalize treatment and support adolescent patients and their families to make sound decisions about pain management. There are data from a school-based sample of adolescents in Norway that suggest that 26% of those polled used OTC medications daily to weekly^8^ and that many of these adolescents also reported complex intersecting problems such as stress.^9^ To our knowledge, there are no North American data regarding the use of OTC medications specific to adolescents with chronic pain. Thus, the purpose of this study was to explore the use, decision-making process, and communication about the use of OTC medication with HCPs in a multidisciplinary chronic pain team setting in adolescents living with chronic pain and their primary caregiver.

## Materials and methods

### Study design

The study received approval from the Research Ethics Board of the hospital and all participants provided free and informed written consent. A prospective descriptive qualitative design was used with semistructured interviews of adolescents with chronic pain and, if present, their primary caregiver (parents, legal guardian, etc.). Interviews were conducted to understand the use of OTC oral and topical pain treatments. Many young people with chronic pain manage their pain with their primary care physician in the community and a smaller proportion are referred to specialized multidisciplinary pain treatment centers within tertiary care centers.^10,11^ The design used phenomenological inquiry, which encompasses qualitative approaches to inductively and holistically understand a human phenomenon (i.e., use of OTC medications to manage pain) in a context-specific setting (i.e., in this case a multidisciplinary tertiary care centre).^12^ NVivo software was used to identify themes relevant to study questions.^13^ Qualitative methods were selected because they provide an opportunity to ask probing questions, uncover subthemes within a semistructured interview, and yield richer data than a quantitative approach. The adolescent and parent were given the choice to have the interviews conducted together (as a dyad), concurrently in separate interviews, or consecutively, based on their preferences.

### Participant recruitment strategy

A convenience sample of individual patients and/or patient–caregiver dyads was recruited from one large tertiary pediatric care center in Southern Ontario, Canada, during their regular follow-up visits to a multidisciplinary chronic pain clinic. The health team at the clinic includes anesthesiologists, advanced practice nurses, psychologists, a psychiatrist, and physiotherapists. Patients referred to the clinic typically include individuals with chronic pain lasting more than 3 months who have shown poor clinical response to conventional pain management therapies.

### Adolescent and caregiver selection

Adolescents were eligible for the study if they were (1) aged 12–18 years, (2) diagnosed with chronic pain, and (3) able to speak and read English. No restrictions were imposed in relation to diagnosis to ensure diverse perspectives from adolescents living with chronic pain. Adolescents were excluded if they had (1) severe cognitive impairments, as determined by their health care team, or (2) major comorbid medical or major psychiatric illnesses (e.g., severe anxiety disorder), which could preclude their ability to participate in a verbal interview as per their HCP. Primary caregivers were eligible for the study if (1) their child met all of the inclusion criteria and none of the exclusion criteria and consented/assented to participate and (2) they were able to speak and read English.

### Interview protocol

Informed written consent was obtained prior to each interview. Participants then completed a short questionnaire on demographic characteristics as well as chronic pain and medication-related information. Interviews were conducted until data saturation was achieved, as confirmed by consensus from analysts that all transcribed data fit into existing subthemes and no new information was emerging that would necessitate new subthemes. All participants chose to be interviewed together as an adolescent–parent dyad, with the exception of one adolescent who did not have a caregiver present. Interviews lasted between 15 and 26 min (mean = 18 min; *SD* = 3.9 min). Interviews were conducted during a mutually convenient time in a quiet meeting room. All interviews were audio-recorded and field notes were written following the interview to document the interviewer’s impression of participant responses and comfort level during the interview.

A semistructured interview guide was used to facilitate each individual session (see Table 1). To begin, a general introductory question was asked (e.g., “Tell us a little bit about the type of pain you have and how long you have had it”). This introduction was followed by more specific questions about OTC medications (e.g., “Tell us about any pain medications that you have tried that you applied directly on your skin”). Probing questions (e.g., “How did it help or not help?”) were used to encourage adolescents–caregivers to elaborate on their experiences with OTC medications for chronic pain. Examples of questions used in the individual semistructured adolescent interviews are presented in Table 1. Design of the interview guide is based upon our team’s collective experience in the management of pediatric chronic pain, through a literature search, and from our previous work.^14^ Questions moved from general to more specific with the aim of addressing the research question by surveying the use of OTC medication in adolescents with chronic pain, how they make decisions to use these medications, and how they communicate this use to their health care team. Individual interviews were conducted by two team members who were trained in qualitative interview techniques (EG and LH). The team members utilized interviewing techniques to minimize the power differential between the interviewers and respondents, such as active listening and relaxed body language.^15^ The probes used were modified during the course of the study in light of emerging themes. At the end of the session, adolescents and caregivers were provided with a small honorarium to thank them for their participation.

**Table 1.** Individual semistructured adolescent interview guide.

Broad question	Probing follow-up question(s)
1. Tell us a little bit about the type of pain you have and how long you have had it.	n/a
2. Tell us about any over-the-counter pain medications or alternative medicines that you have taken by mouth and you have used to help with your pain.	What ones have you used? Do you remember specific brands or names? How did it help or not help? Have you experienced any side effects using these over the counter pain medications? If so, what kind of side effects? Was there anything in particular that you liked or did not like about over-the-counter medications you have tried? Did you feel like you know how much of the over-the-counter medication to take (or the dose)?
3. (a) Tell us about any pain medications that you have tried that you applied directly on your skin.	Some potential topical applications as probes: Essential oils, clove oil, rutin, camphor, eucalyptus oil, menthol, heat, ice gels, patches, wraps, *Arnica*, NSAID/diclofinac creams, and ointments
(b) If you have not used topical pain treatments, or only use oral pain treatments, why have you not considered a topical analgesic treatment option?	When did you start using it? How often did you use it (dose)? Did you use it on its own or in combination with other over-the-counter medications? What was your experience with that medication? How did it help or not help? Did you experience any side effects using these pain treatments? If so, what kind? Was there anything in particular that you liked or did not like about the over-the-counter topical pain treatments?
4. What decision-making process did you use to select specific oral or topical pain treatment options?	Is price a factor when you are choosing which pain treatment to purchase?
	Does your parent make the decision in choosing which over the counter pain treatment to buy or do you make the decision on your own?
	Did anyone suggest or recommend over-the-counter treatments? If so, who?
	Is there a certain brand name that you prefer to purchase? If so, which brand(s) is it?
	Have you purchased a specific over-the-counter oral or topical pain treatment upon a recommendation from a member of your health care team (doctor, nurse, physical therapist, psychologist)?
	Did concern about possible drug–drug interactions factor into your decision? If so, can you tell me about those concerns?
5. What types of pain have you used oral and topical pain treatments to manage?	Certain body parts?
	Certain types of pain—chronic or acute pain? Nerve pain or muscle pain?
6. Would you recommend oral and or topical pain treatments to other patients?	Why or why not?
7. Do you feel more comfortable using over-the-counter or alternative medicines than prescription medicines?	If so, why?
8. How do you communicate with your doctor or health care provider about using these over-the-counter pain therapies?	Do you have any concerns about talking to them about using these products? If so can you tell me about them?
9. Is there anything else you wanted to tell us about their use that we did not discuss?	n/a

NSAID = nonsteroidal anti-inflammatory drug.

### Data analysis

All interviews were transcribed verbatim and then verified against the tapes. Transcripts were then imported into NVivo 10 for Windows, a qualitative software program that allows for data organization, coding, and retrieval.^12^ Field notes taken after the interviews were also included in the analytical process. NVivo 10 was used to carry out simple content analysis.^16^ Two researchers (EG and LH) coded the interviews separately using NVivo 10. Disagreement surrounding phrasing of themes was handled through consensus and intersubjective agreement of both researchers. Qualitative simple content analysis was used to guide the organization of data into nodes (NVivo categories) that reflected emerging themes. Parent and adolescent data were analyzed together as dyads; however, as themes emerged, the data were revisited to identify any differences between adolescent and parent perspectives on each topic. The raw data were revisited frequently throughout the analysis to ensure validity and accuracy of emerging themes.^17^

## Results

### Study participants

Demographic characteristics of the adolescent and caregiver sample are summarized in Tables 2 and 3, respectively. A total of 15 adolescents and 16 caregivers participated in the parent–adolescent interviews. Two adolescents had both parents participate, and one adolescent participated without a caregiver present. The recruitment period lasted from October 2014 to March 2015. Twenty adolescents were approached and invited to participate, of whom 15 agreed to participate (75% recruitment rate). Reasons for not participating included unable to make 1-h time commitment (*n* = 2), not interested (*n* = 2), or not eligible due to language barrier (*n* = 1).

**Table 2.** Demographic characteristics of the adolescent sample, *N* = 15.

Characteristic	Results
Current level of education, *n* (%)	
Grade 8	3 (20%)
Grade 9	0 (0)
Grade 10	3 (20%)
Grade 11	3 (20%)
Grade 12	6 (40%)
Age (years), mean ± SD	16 ± 1.8
Sex, *n* (%)	
Female	12 (80%)
Male	3 (20%)
Race, *n* (%)	
White (Caucasian)	9 (60%)
Asian	3 (20%)
Black Canadian	1 (7%)
Mixed	2 (13%)
Types of chronic pain,^a^ *n* (%)	
Neuropathic	7 (47%)
Complex widespread	3 (20%)
Complex regional pain syndrome	4 (27%)
Other	7 (47%)
Undiagnosed	2 (13%)
Duration of pain (months), mean ± SD	45 ± 50.4
Number of medications,^b^ mean ± SD	3 ± 1.6

^a^Some participants listed more than one type of chronic pain.^b^medications include both over-the-counter and prescribed. Only two participants listed over-the-counter medications in the demographics list; however, most described using them in the qualitative interview. This table reflects the quantitative survey data only.

**Table 3.** Demographics of the caregiver sample.^a^

Characteristic	Caregivers (*n* = 16)^b^
Age (years)	
30–39	1 (6)
40–49	9 (56)
50–59	5 (31)
Gender	
Female	12 (75)
Male	3 (19)
Marital status	
Married	15 (94)
Single	1 (6)
Highest level of education	
High school	1 (6)
College/university	13 (81)
Professional/graduate degree	2 (13)
Annual income ($)	
0–29 999	3 (19)
30 000–59 999	2 (12.5)
60 000–89 999	2 (12.5)
>90 000	8 (50)
Prefer not to disclose	1 (6)

^a^Data presented as *n* (%) unless otherwise indicated.

^b^Demographic data were not completed for one caregiver.

### Thematic analysis

**Figure 1.**
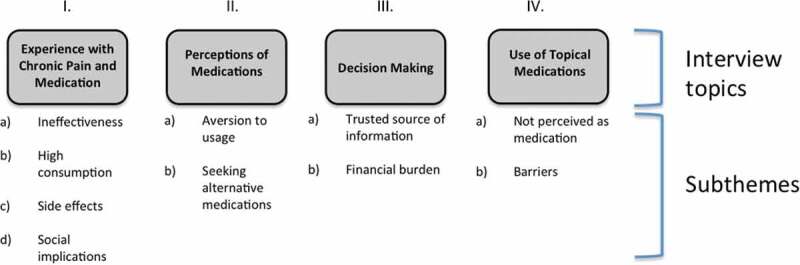
Coding scheme for adolescent and caregiver interviews.

Major topics from the semistructured interview guide was used to categorize the data as follows: (1) experience with chronic pain and OTC medication; (2) perceptions of medications; (3) decision making about OTC usage; and (4) use of topical OTC medications. Under each topic, there were two to four subthemes that will be explored in detail below. Responses were consistent between adolescents and their caregivers. See Figure 1 for the complete coding scheme.

#### Experience with chronic pain and OTC medications

This topic describes the experiences of adolescents with using OTC medications to manage their chronic pain and includes four subthemes, described below.

##### Ineffectiveness of OTC medications for chronic pain

Almost all adolescents and their caregivers agreed on the ineffectiveness of OTC medications for the treatment of chronic pain. One adolescent said, “… We’ve kind of figured out that they don’t help with that [neuropathic pain]. I don’t think they’re strong enough and they’re not built the right way to help with that” (female, age 16). Another adolescent stated, “If you have really bad pain, don’t take Tylenol thinking that it will help your pain cause it probably won’t but it can help your headache or something” (female, age 18). When discussing trying diclofenac for his complex regional pain syndrome, one adolescent said “… It didn’t do anything. I stopped it” (male, age 16). Parents/caregivers described trying OTCs when they felt they had no other options, as evidenced by one mother’s quote: “We have tried Advil occasionally when she’s felt really desperate, but it’s useless.”

##### High consumption of OTC medications

Despite reporting that OTC medications are generally ineffective, the majority of adolescents in this study reported regular consumption of OTC medications. In most cases, participants reported taking regular doses within the manufacturer’s recommended daily dose as needed. The types of OTC medications listed by participants are outlined in Table 4. One mother described: “The over-the-counter stuff, it’s really good in really general situations but it could be really easy to overmedicate somebody who suffers chronic pain with the over-the-counter stuff, especially if she’s not getting the benefit from it.” Similarly, one adolescent stated, “I was taking them a lot for nine months or ten months and it was really a lot, like around the clock and so the doctors thought that wasn’t really healthy like for my stomach” (female, age 12). Another adolescent described a similar situation: “By the time I got my nerve disorder I had already been taking Tylenol every day for five years” (female, age 17). In one case, safety concerns were noted and addressed by the interviewer, when the adolescent reported taking more than the recommended dose.

**Table 4.** OTC medications used by the study participants, as described in qualitative interviews.

Generic medication (brand)	Method of application	No. of participants (*n* = 16, %)
Acetaminophen (Tylenol)	Oral	13 (87)
Ibuprofen (Advil)	Oral	6 (40)
Dimenhydrinate (Gravol)	Oral	5 (33)
Diclofenac (Voltaren gel)	Topical	3 (20)
Diphenhydramine (Benadryl)	Oral	2 (13)
Melatonin	Oral	2 (13)
Naproxen (Aleve)	Oral	1 (7)
Menthol (Biofreeze)	Topical	1 (7)

OTC = over-the-counter.

##### Side effects

All of the adolescents described the side effects they have experienced from using OTC medications. A common side effect discussed from dimenhydrinate (Gravol) was drowsiness (*n* = 5 participants; 33%), especially as it affected their school engagement: “It’s the side effects. That’s what worries me the most cause it’s hard being drowsy when you’re at school because you can’t focus” (female, age 17). Adolescents described limiting their daytime use of OTC medications that can cause drowsiness in order to avoid this disruptive side effect during school hours, and one adolescent described seeking nondrowsy versions of the OTC medication. When asked what they do to manage their nausea during the day, one adolescent stated, “Nothing really. I just have to deal with it” (female, age 15). In addition to drowsiness, several adolescents described stomach upset as a side effect from OTC medication. As one participant described, “They say that Advil is not supposed to be hard on the stomach but it irritates my stomach” (female, age 15). Some patients made the decision to stop a medication because of the side effects. They went on to say “Sometimes I’m like, ‘I’m getting off this [Tylenol] even if my hip hurts I don’t want to get another issue,’” referencing the side effects such as nausea that she had experienced from the OTC medication.

##### Social implications

Some adolescents described the social implications of using OTC medications in school. The primary concern was with the smell that accompanies OTC topical medication. One mother described her 14-year-old’s reluctance to use topical OTC medications before school: “Yeah, she likes to put it on at night but not in the morning because she doesn’t want to go smelling in the school, you know?”

#### Perceptions of prescribed and OTC medications

This topic describes the perceptions that adolescents and their caregivers expressed toward taking medication in general, including both OTC and prescribed medications. Two subthemes are described below.

##### Aversion to usage

Most of the participants, both adolescents and caregivers, demonstrated an aversion or dislike of taking medications in general, including both OTC and prescribed medication. Adolescents stated that their primary concerns were the health implications of long-term medication usage, as well as the unknown effects of medications in their bodies. “You’re just putting stuff [medication] into your body that’s not working, and just because you don’t feel any side effect doesn’t mean there’s nothing right?” (female, age 17). Another adolescent voiced her position: “I’m not someone going out to [seek out new] medications because I don’t want to use them. Like I want to use them as little as possible” (female, age 15).

Parents mirrored their adolescents’ concerns, yet were conflicted with the desire to reduce their child’s pain with medications if possible. As one father stated, “We try not to use over-the-counter medications generally, but we know we have to so we limit the use where possible.” Another mother said,

I’ve never been a big fan of medications period, so I have a hard time with—even with the over-the-counter medications and stuff. I just don’t like her being on all that medication. But what else, we were just trying everything, you know?

One mother sums up the subtheme by stating, “Ideally it’s no meds but the reality is we are here.”

##### Seeking alternative medications

In addition to an aversion to taking OTC or prescribed medication, a common theme discussed was the perception of using complementary and alternative medications (CAMs) to help with pain and the frequency of their use. One adolescent stated: “I wanted to see if there was something else other than the prescribed stuff” (female, age 18). CAMs were generally not perceived as “true” medications by most participants. This perception was exemplified by one mother who dismissed the CAM product they tried as follows: “It’s *just* an herbal medicine.” It was common for adolescents to have tried alternative medications based on the recommendation of family or friends. Although 10 of 15 participants reported the use of a CAM, 4 of those 10 could not remember the name of the alternative medication used. For example, when asked by the interviewer whether the oil-based product they apply had any medicinal ingredients they stated, “I doubt it” and “No, it’s just a very natural [product]” (female, age 18).

#### Decision making

This topic describes the factors considered when making decisions around medications, including which new OTC medications to try and when to take OTC medication. The two subthemes identified in this study include consultation with a trusted and accessible source of information, as well as financial implications.

##### Trusted source of information

Decision making about the use of OTC medications was consistently made in conjunction with a trusted source of information, in most cases a HCP. Although many of the participants were taking medications independently, all reported in the joint parent–teen interview that they would still tell their parent/caregivers when they did take an oral OTC medication. Most adolescents described the decision making about OTC medications as a “joint decision” (female, age 15) between themselves, their parent, and their health care team. Six (40%) of the participants reported that they do not first consult with their HCPs when taking acetaminophen or nonsteroidal anti-inflammatory drugs (NSAIDs), for example. Seven (47%) participants reported that they do consult their HCP (either primary care providers such as family doctor or HCPs within the pain clinic), and the other two stated that they would “probably” go to a family doctor or pharmacist. When asked whether they ever had concerns about talking about OTC medications or CAM regimens with their HCPs, all participants stated that they do not have this concern. However, when probed further, many adolescents and parents thought that their use of OTC medications was not relevant for their HCP to know. In terms of which HCPs were consulted, pharmacists were consistently mentioned as trusted and accessible sources of information about OTC medications. For advice between health care appointments, more than half of the adolescents (*n* = 8; 53%) discussed reaching out to relatives or family friends for OTC or CAM recommendations. Three of those eight mentioned were regulated HCPs (MD, RN, and PT); however, none were actually on the care team of the adolescent.

##### Financial burden

Caregivers bore the financial burden of OTC medications for their adolescent children. “Price is a huge factor for sure,” said one mother. Four out of 15 families reported price to be a factor when purchasing OTC medications. Although most families were aware of the expense of OTC medications, the participants were evenly split on brand name versus generic; half of the dyads would buy the generic name over the brand name based on cost (“If it’s the same ingredients, why would I pay more money for something that says [a name brand]?” [female, age 17]; one mother stated: “It’s supposed to be the same material in it but it costs less cause it’s, you know, you’re not paying for the advertising and such”). The other half of respondents reported that they would buy brand name over generic because they trust the brands; as one mother explained: “I don’t know what’s in the other stuff.” One dyad was indifferent about cost. Additionally, one family (7%) noted that prescription medications are more likely to be covered by their insurance policy and preferred to use prescription whenever possible.

#### Use of topical OTC medications

The focus of this topic is the way that topical medications are perceived by adolescents with chronic pain and their caregivers. This topic describes how they are perceived differently from oral medications and also addresses barriers to using topical medications that were discussed.

##### Perceived differences between oral and topical OTC medications

The usage and efficacy of topical medications varied widely among this sample. Only two (13%) of the participants voiced concerns with medication interaction between topical and oral medications. Another participant had knowledge of medication interactions between different topical medications. When asked about concerns with topical–oral medication interaction, one mother said, “To be honest I see it as two different things,” and an adolescent responded, “I just think it’s a cream” (female, age 18). Generally, topical medications were not treated with the same degree of caution as oral OTC medications. As one father described:

I think it depends on what the product is you’re talking about, is it something like a topical patch or something like that she would use her best judgment but if it’s something in which she has to ingest it then she would definitely contact the [pain] team.

##### Barriers to use of topical OTC medications

Most participants reported significant barriers to the usage of topical medications. Common barriers included the texture or smell of the medication, as well as the burning sensation when applied. Many participants reported burning and painful application as one of the main reasons they do not use topical medications: “Burns like it shouldn’t be on my skin” (female, age 18). In particular, participants with neuropathic pain conditions were unable to use topical creams that create heating or cooling effects because they would actually increase pain. Other significant barriers included difficulty of application, specifically back areas: “That’s not the easiest place to put it on yourself” (female, age 17), as well as short duration of effect; as one mother stated, “It was only good for ten minutes at time.”

## Discussion

The aim of this study was to better understand how youth with chronic pain use OTC medications, make decisions about their use, and communicate with HCPs about OTC medications. This research focus is crucial in order to help clinicians better personalize treatments and support adolescents with chronic pain to make sound decisions about pain management. This study uncovered that adolescents report a high frequency of OTC medication usage, yet they perceive the ineffectiveness of these medications as a way of helping to manage their pain. They report an aversion to taking medication in general and typically consult either their own HCP or family members as a trusted source of information about medications. This study also found that adolescents and their parents perceive topical medications as harmless compared to oral medications.

Youth in this study and their parents described their experiences using medications for chronic pain, including regular consumption of OTC medications despite their perceived ineffectiveness. The results suggest that adolescents continue to take OTC medications despite ineffectiveness because they feel desperate; as one parent stated: “I just don’t like her being on all that medication. But what else, we were just trying everything, you know.” Adolescents described their experiences managing side effects and navigating the negative social implications of using OTC medications at school. The high-frequency consumption of OTC medications is consistent with other recent findings. According to one Norwegian study,^6^ over one quarter of teens surveyed from a community-based sample (aged 15–16) had used OTC medications daily or weekly within the past 4 weeks. Not surprisingly, those with high-frequency use more often reported pain in several body parts. Additionally, those high-frequency users reported less sleep, higher consumption of caffeinated drinks, and lower self-esteem. Another high school–aged sample from the United States reported that 85% of teens used OTC medications as a pain self-treatment method.^18^ One hypothesis suggested by Skarstein and colleagues is that the high-frequency consumption of OTC medications might be reflective of a compromised ability to handle stress.^8^ Though this particular study included teens with and without chronic pain, an area of future research should focus on how a lack of effective coping skills may correlate to the high use of OTC medications. There has also been some speculation that acetaminophen may have anxiolytic-related properties,^19,20^ although no definitive evidence exists to support this hypothesis. Further research is needed to evaluate whether acetaminophen may play a role in managing adolescents’ anxiety.

Given the extensive use of OTC pain medications among young people, it is critical that patient education is tailored to provide accurate and accessible information to young people about the risks, benefits, and potential side effects of high-frequency use of OTC medications. For example, overuse of OTC medication can cause headaches, known as “medication overuse headache.”^6^ Another study by Westergaard et al. showed that more than half of their sample surveyed who have chronic headaches also have concurrent OTC and/or prescription medication overuse.^21^ Though this result is correlational, patients who take OTC medications regularly for a particular chronic pain complaint may be at risk for developing headaches in addition to their primary pain. Improved patient education, along with targeted questions from the HCP, could potentially reduce the incidence of negative side effects such as OTC medication overuse headache.

In addition to OTC usage, many adolescents reported trying CAMs to help manage their pain. Notably, patients did not perceive CAMs as true medications, and many did not know whether they contained medicinal ingredients. A number of reasons were provided by this sample for using CAMs, including having received recommendations from a trusted source and a desire to try something other than their prescribed medication. A Canadian national survey of adults with inflammatory bowel disease, a painful condition, concluded that the decision to try CAMs is tied to disease activity and health attitudes and behaviors, rather than demographic information.^22^ Another Scandinavian study reported specific reasons that patients with inflammatory bowel disease cited for using CAMs included a lack of satisfaction with conventional therapies including their side effects and the perceived safety of CAM relative to traditional medications.^23^ Patient education should focus on communicating safe practices with selecting and using CAMs for chronic pain management.

Consistently, both adolescents and their parents described a dislike of frequent OTC medication consumption; however, they felt that the adolescent had no other viable pain management options. The most common concern about medication that was noted by participants in this study was the general sense of unease regarding putting medications into their bodies, particularly if they did not perceive a benefit (i.e., pain relief). Additionally, many sought to include CAMs in their pain management plan.

Topics 1 and 2 together highlight the conflict that is faced by adolescents with chronic pain and their caregivers about taking OTC pain medications. Though they consistently reported that they did not like using medications, they also felt that they did not have other viable options for managing their chronic pain. We found that despite a clear dislike of long-term OTC medication consumption, this population continues to regularly ingest and apply OTC medications within their chronic pain management plan. Furthermore, this sample has access to a multidisciplinary tertiary care clinic for chronic pain, so it is conceivable that those adolescents living with chronic pain without access to a multidisciplinary team could rely more heavily on OTC and prescribed medications as a primary pain management strategy. The frequent use of OTC medications despite ineffectiveness highlights the need for improved pain management education and the critical role of self-management in chronic pain management.

Based on these joint parent–adolescent interviews, it was reported that adolescents and their parents made joint decisions regarding the use of OTC medications; however, the financial burden fell primarily on the parents. Notably, half of our sample was in a high-income bracket (see Table 3) and it would be valuable in future studies to better understand how household income influences decisions about OTC usage. Based on the findings from this study, adolescents reported that they had no concerns with talking to their HCPs about their OTC medications/CAM regimens; however, they often thought of it as not being relevant for their HCP to know. This finding is consistent with another recent qualitative study, in which adult participants with chronic pain were quoted as describing OTC medication as “just over-the-counter” and “not real pain medication” (ref. 24, p. 149). In that same study, the majority of participants reported that they did not take pain medication; however, when probed, in fact they did take OTC medication but did not consider OTC as “pain medication” when asked originally. Therefore, HCPs who work with adolescents with chronic pain should ask targeted questions to better understand whether and how their patients are using OTC and CAM. Adolescents in this study reported using OTC medications after they stopped being effective; thus, HCPs could better educate patients about how long to trial OTC medications and, if they do not work, to speak with their HCP about other options. The health care team should also provide information about correct dosage if patients are using OTC medications inappropriately.^25^

Finally, both adolescents and their parents perceived and treated topical OTC pain medications differently than oral OTC medications in terms of safety and risk. A similar result was found in a study with older adults with heterogeneous knee pain, where the adults reported that they preferred to use oral NSAIDs to treat pain compared to topical NSAIDs.^26^ Further, they reported concerns about the side effects of oral NSAIDs but generally had not considered the possibility of topical medications having similar negative side effects. Interestingly, the overall safety and efficacy of topical NSAIDs compared to oral therapy remains controversial. Roth and Fuller found that topical and oral diclofenac were equally effective for adults with osteoarthritis but that the topical preparations had fewer side effects, presumably due to minimal systemic absorption.^27^ Currently, no guidelines exist on the use of combination oral and topical NSAID therapy. Clinical implications of these findings include the need to develop patient education materials related to OTC medications, specifically regarding the mechanisms of action, contraindications, and potential side effects for each product. Material developed for public knowledge should be user-friendly and written in accessible language.

## Study limitations

First, all participants were recruited from a tertiary care, multidisciplinary team setting, so it is not known how findings may differ in the context of primary care or other tertiary care settings. Thus, these results cannot be generalized to a primary care setting, and future research should focus on identifying whether the themes identified at our site related to OTC use in adolescents with chronic pain are applicable within other patient populations in order to best inform and tailor patient education materials. At this site, data saturation was achieved by interviewing 15 patients and 16 caregivers, suggesting that similar studies would be feasible to conduct across multiple sites, such as different countries and cultures, primary care settings, or younger age groups with chronic pain. Second, the majority of the patients recruited were female, with only three males interviewed. This result is not surprising because the prevalence of chronic pain is significantly higher in females. However, there have been reported sex differences in terms of likelihood to use both OTC medications and nonpharmacological strategies for pain (females are more likely to use both).^16^ Potential cultural differences in pain perception and management should also be considered,^28^ given that 60% of the participants in this study were Caucasian. Third, all adolescents accompanied to the appointment by a parent opted to complete the interview together with their parent. The presence of their parent may have affected adolescent self-reporting of medication usage. Future studies that collect information from adolescents independently from their parents might provide further insight into adolescents’ decision making around use of OTC medications. Finally, this study did not collect information regarding the use of prescription medications. Future studies that collect this information could explore the relationship between use of OTC and prescription medications.

## Conclusion

This study provides insight regarding how adolescents and their parents use and perceive OTC medication within the context of chronic pain management. Because adolescents use OTC medications so frequently, patient education is critically needed to ensure that the specific risks, side effects, and costs of OTC medications are discussed with patients. Future research should focus on the development of effective patient education materials as well as strategies for communicating about OTC medications with HCPs. Finally, future evidence is needed on the effectiveness and adverse effects of OTC medications and on identifying which patients benefit from which products, as measured through scientifically rigorous, blinded controlled trials.
